# Preparative Scale Production of Functional Mouse Aquaporin 4 Using Different Cell-Free Expression Modes

**DOI:** 10.1371/journal.pone.0012972

**Published:** 2010-09-24

**Authors:** Lei Kai, Ralf Kaldenhoff, Jiazhang Lian, Xiangcheng Zhu, Volker Dötsch, Frank Bernhard, Peilin Cen, Zhinan Xu

**Affiliations:** 1 Department of Chemical and Biological Engineering, Institute of Biological Engineering, Zhejiang University, Hangzhou, China; 2 Centre for Biomolecular Magnetic Resonance, Institute for Biophysical Chemistry, Goethe-University of Frankfurt/Main, Frankfurt/Main, Germany; 3 Institute of Botany, Technical University Darmstadt, Darmstadt, Germany; University of Crete, Greece

## Abstract

The continuous progress in the structural and functional characterization of aquaporins increasingly attracts attention to study their roles in certain mammalian diseases. Although several structures of aquaporins have already been solved by crystallization, the challenge of producing sufficient amounts of functional proteins still remains. CF (cell free) expression has emerged in recent times as a promising alternative option in order to synthesize large quantities of membrane proteins, and the focus of this report was to evaluate the potential of this technique for the production of eukaryotic aquaporins. We have selected the mouse aquaporin 4 as a representative of mammalian aquaporins. The protein was synthesized in an *E. coli* extract based cell-free system with two different expression modes, and the efficiencies of two modes were compared. In both, the P-CF (cell-free membrane protein expression as precipitate) mode generating initial aquaporin precipitates as well as in the D-CF (cell-free membrane protein expression in presence of detergent) mode, generating directly detergent solubilized samples, we were able to obtain mg amounts of protein per ml of cell-free reaction. Purified aquaporin samples solubilized in different detergents were reconstituted into liposomes, and analyzed for the water channel activity. The calculated *P*_f_ value of proteoliposome samples isolated from the D-CF mode was 133 µm/s at 10°C, which was 5 times higher as that of the control. A reversible inhibitory effect of mercury chloride was observed, which is consistent with previous observations of *in vitro* reconstituted aquaporin 4. In this study, a fast and convenient protocol was established for functional expression of aquaporins, which could serve as basis for further applications such as water filtration.

## Introduction

Aquaporins (AQPs) are a family of channels permeable to water and some other small solutes such as glycerol and urea. Characterized by six transmembrane segments and two loops that also are embedded into the membrane, AQPs are abundant in all kinds of organisms from bacteria to higher eukaryotes [1]. Among them, AQP4 is known as a water-transporting aquaporin with two isoforms differing from their N-termini, which result from variable translation starting sites either from methionine M1 (323 aa) or methionine M23 (301 aa) [2]. AQP4 is found in many organs and tissues like kidney, skeletal muscle, stomach, brain, and lung airway epithelium. Phenotype analysis of AQP4-knockout mice indicated its involvement in the brain water balance [3]. AQP4 expression was furthermore shown to be induced during spongiform encephalopathy [4], neuromyelitis optica, multiple sclerosis [5], and Alzheimer's disease [6]. In addition, AQP4 provides a molecular pathway for water permeability and homeostasis in the brain, and its astrocytic end-feet localization makes AQP4 a partner to blood-brain-barrier function [7].

Sufficient amounts of protein have been obtained for 2D crystallization and functional characterization of rat AQP4 [8]. Nevertheless, the purification processes are time-consuming and the yield with approx. 3 mg/l produced in insect cells [8] is still not satisfying if compared with that of soluble proteins. Further biochemical and biophysical characterizations as well as high-throughput research towards the relationship between aquaporin and certain diseases remain therefore to be difficult. Recently, an increasing interest is to prepare some functional biomembranes integrated with bioactive AQPs to filtrate water [9]. However, the success of this strategy largely depends on the availability of sufficient and functional AQPs, which still remains challenging [10], [11].

CF expression systems have been developed in the early 1950s initially as batch systems with low yields of recombinant proteins in nanogram or microgram scales [12], [13], [14]. However, with the development of CECF (continuous exchange cell-free) system milligram scales of recombinant protein could be produced overnight [15]. In this more efficient CF expression system, a RM (reaction mixture) containing all the high molecular weight compounds of the reaction is separated by a semi-permeable membrane from a FM (feeding mixture), providing fresh low molecular weight precursors for the reaction.

CECF expression systems based on *E. coli* cell extracts have recently been demonstrated to provide a new and highly promising tool for the preparative scale production of membrane proteins [16], [17], [18]. Besides the elimination of toxic effects, a unique advantage of CECF systems is the possibility of directly producing soluble membrane proteins in the presence of detergents [16], [19]. In this D-CF mode of expression, the synthesized membrane proteins will be solubilized co-translationally or shortly after translation by the supplied detergent micelles. In the alternative P-CF (precipitate forming) expression mode, no hydrophobic environment is provided and the synthesized membrane proteins will quantitatively precipitate in the RM. These precipitates can readily be solubilized in detergents without prior denaturation/renaturation steps and functionally folded membrane proteins may be obtained even with complex targets [18], [20]. Hence, both D-CF and P-CF strategies provide feasible ways to produce membrane proteins solubilized into detergent micelles [15], [21].

To address the challenge of producing sufficient amounts of functional aquaporins, the expression efficiency of an individual *E. coli* based CECF system via the D-CF and P-CF mode was evaluated, respectively. The shorter derivative of the mouse water specific aquaporin 4 (mAQP4 M23) was selected as the candidate. As a result, the mAQP4 M23 is the first eukaryotic aquaporin produced in preparative scales through CF systems, and we have been able to synthesize up to 2 mg protein per one ml of RM. We have also demonstrated that both P-CF and D-CF modes are able to produce functionally folded mAQP4 M23. A fast and convenient protocol was established, which takes no more than two days to start from the set-up of reaction to the final assay of water channel activity.

## Results

### Development of cell-free expression protocols for the preparative scale production of mAQP4 M23

Previously described CECF reaction conditions were used as criteria for the expression protocol development [22]. The first 22 codons are not essential for the mAQP4 water transport activity and thus were deleted in our construct [23], [24]. The mAQP4 M23 coding sequence was cloned into the vector pIVEX2.3MCS and the template was designed for the production of a modified mAQP4 M23 containing an additional 12 amino acid N-terminal T7-tag as well as a poly(His)_10_ purification-tag at the C-terminal end. The calculated molecular mass of this mAQP4 M23 derivative is 30 kDa. Critical parameters for CF protein production are optimal ion concentrations in particular of Mg^2+^ and K^+^. After screening of Mg^2+^ and K^+^ ion concentrations in the P-CF expression mode in a range between 7–25 mM and 200–400 mM, respectively, optima were determined at 17–22 mM Mg^2+^ and 250–340 mM K^+^. With these conditions, the yield of CF produced mAQP4 M23 in 50 µl analytical scale micro-reactor reactions as well as in 3 ml preparative scale maxi-reactor reactions were routinely in the range of 1.5–2 mg protein per one ml RM.

### Production of mAQP4 M23 proteomicelles in the P-CF and D-CF expression modes

**Figure 1:**
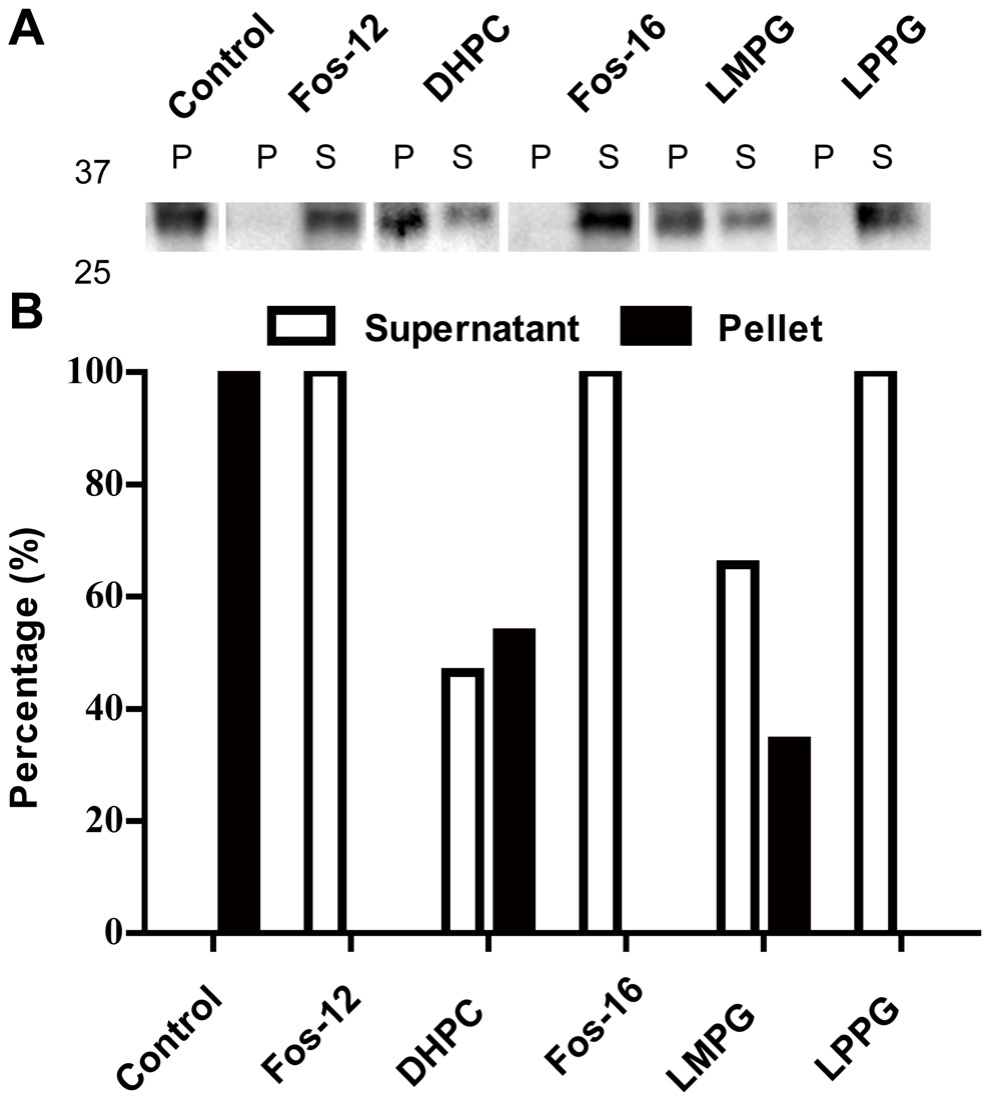
Resolubilization screening of P-CF produced mAQP4 M23. The pellet from the P-CF reaction mix was resuspended with either 1% (w/v) Fos-12, 2% (w/v) DHPC, 2% (w/v) Fos-16, 2% (w/v) LMPG, or 1% (w/v) LPPG. Sample volumes of 4 µl were analyzed by 16% SDS-PAGE. The solubilization efficiency was determined by densitometry after immunoblotting using anti-His antibodies. Control is P-CF expressed mAQP4 M23. A: immunoblotting using anti-His antibodies. S, supernatant; P, pellet. B: The solubilization efficiency determined by densitometry of the immunoblotting.

In the P-CF mode, the mAQP4 M23 precipitated directly after translation within the RM. The precipitates were harvested by centrifugation, washed once with resolubilization buffer (20 mM Tris, pH 7.3, 150 mM NaCl) and instantly resuspended in a variety of detergents for resolubilization, with the same volume as the initial RM volume. For this P-CF resolubilization screen, the detergents Fos-12 (dodecylphosphocholine) (1%), DHPC (1,2-diheptanoyl-sn-glycero-3-phosphocholine) (2%), Fos-16 (n-Hexadecylphosphocholine) (2%), LMPG (1-myristoyl-2-hydroxy-sn-glycero-3-[phospho-rac-(1-glycerol)]) (2%) and LPPG (1-palmitoyl-2-hydroxy-sn-glycero-3-[phospho-rac-(1-glycerol)]) (1%) were evaluated. The initial suspensions were incubated at 37°C for 1 hour with gentle shaking to allow efficient mAQP4 M23 solubilization. Residual mAQP4 M23 precipitate was then removed by centrifugation at 18,800× g for 10 min. The supernatant and residual pellets were subsequently analysed by SDS-PAGE, western blotting and immunodetection of the C-terminal poly(His)_10_-tag. From tested detergents, 1% Fos-12, 2% Fos-16 and 1% LPPG showed optimal solubilization of the target protein. Nearly all precipitates were redissolved (Fig. 1), whereas 2% LMPG and 2% DHPC could only partially solubilize the mAQP4 M23 precipitates and approx. 50% of the protein still remained non-soluble (Fig. 1).

**Figure 2:**
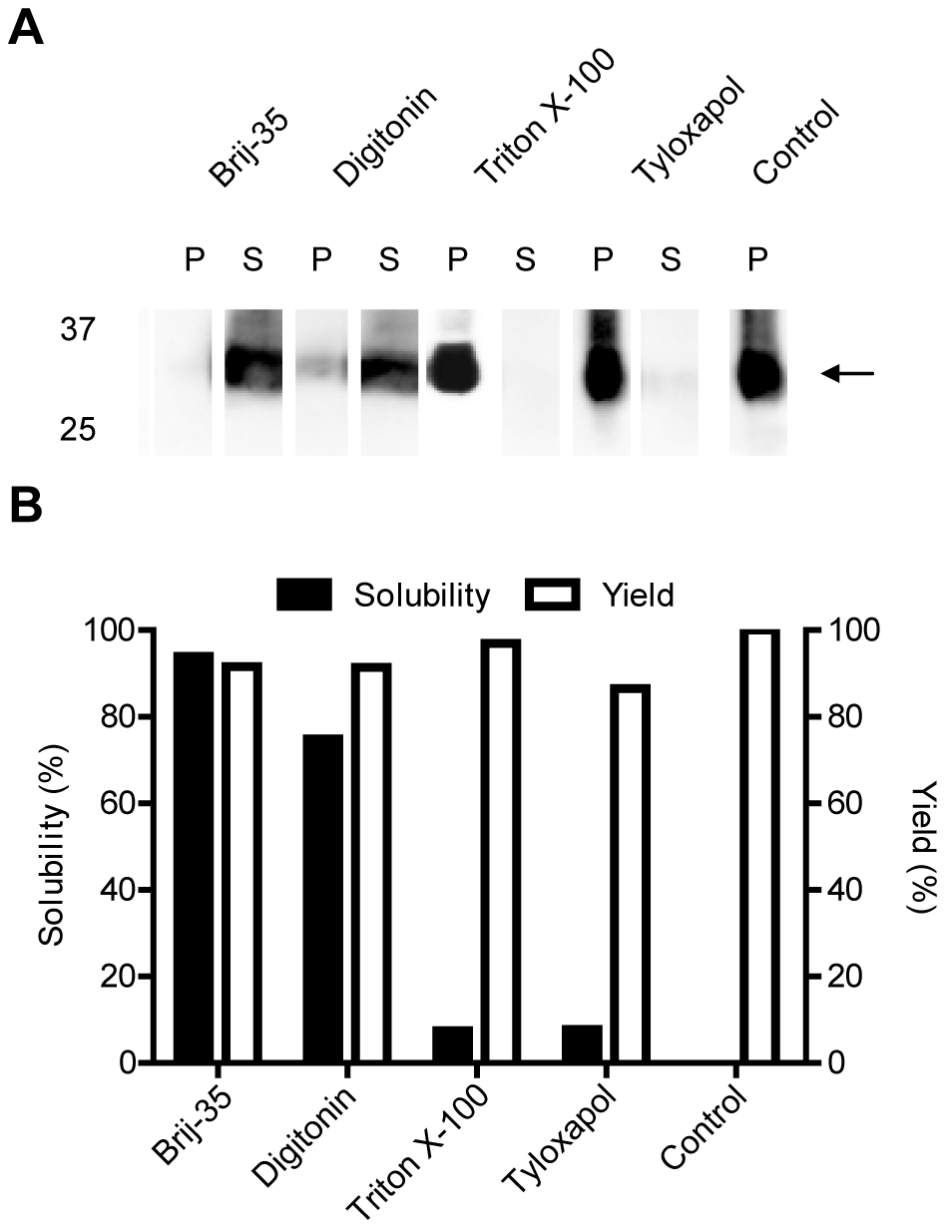
Detergent screening of mAQP4 M23 expressed in the D-CF mode. A: RM samples of 2 µl were analyzed by 16% SDS-PAGE and immunoblotted using anti-His antibodies. B: Solubility of D-CF expressed mAQP4 M23 in presence of 0.2% Brij-35, 0.4% Digitonin, 0.1% Triton X-100, and 0.05% Tyloxapol. Control is P-CF expressed mAQP4 M23. S, supernatant; P, pellet.

Alternatively, soluble mAQP4 M23 was directly produced in the D-CF expression mode. The detergents supplied into the RM provided hydrophobic environments for the co-translational solubilization of CF expressed mAQP4 M23. A number of D-CF suitable detergents including Brij-35 (polyoxyethylene-(23)-lauryl-ether) (0.1%), Digitonin (0.4%), Triton X-100 (0.1%) and Tyloxapol (0.05%) were screened for their efficiency (Fig. 2). In the ideal case, all produced mAQP4 M23 should become soluble while the expression efficiency should not be reduced by the detergent. The detergents Brij-35 and Digitonin showed high efficiency to solubilize mAQP4 M23 without significant effect on the protein expression. With 0.2% Brij-35 in the RM, almost 90% of the expressed mAQP4 M23 was solubilized in proteomicelles. In the presence of 0.4% Digitonin, approximately 75% of synthesized mAQP4 M23 was solubilized. However, Triton X-100 (0.1%) and Tyloxapol (0.05%) were much less effective on the co-translational solubilization of mAQP4 M23, with efficiencies of not more than 10%.

### Purification of mAQP4 M23 and *in vitro* liposome reconstitution

**Figure 3:**
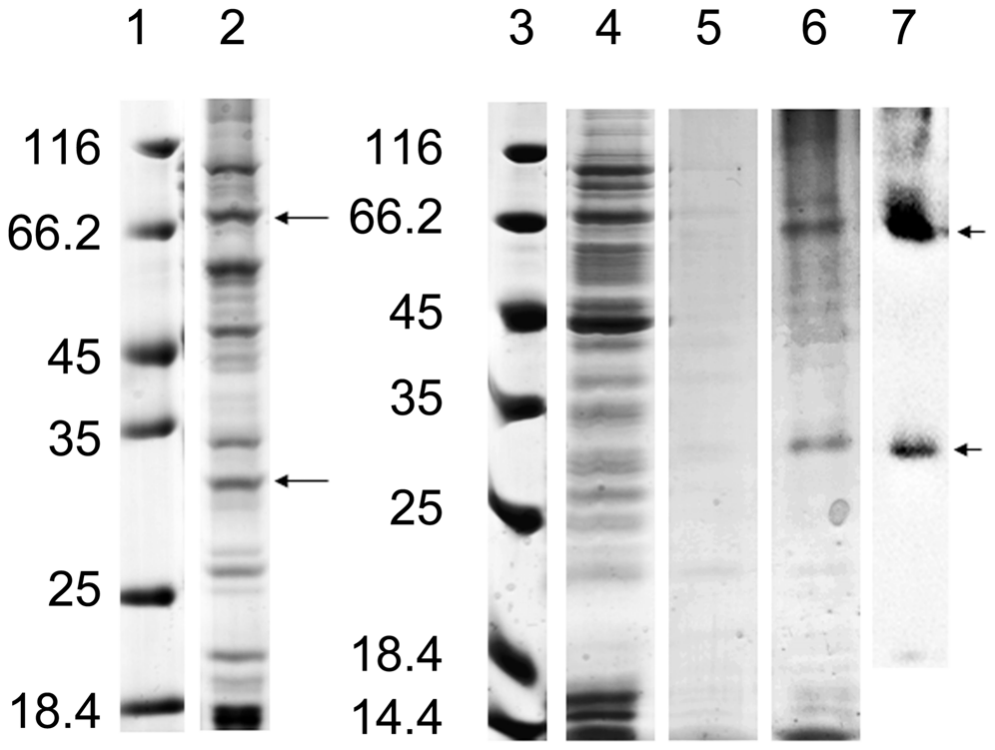
Purification of D-CF produced mAQP4 M23 in 0.2% Brij-35 by Co^2+^-NTA chromatography. Samples were separated by 12% (lanes 1–2) or 16% (lanes 3–7) SDS-PAGE and analysed by Coomassie staining. Lanes 1 and 3, protein marker; Lane 2, precipitate after P-CF expression; Lane 4, flow through; Lane 5, washing fraction; Lane 6, elution fraction; Lane 7, immunoblot of lane 6 using anti-His antibodies. Samples of 2 µl were applied to each lane.

Depending on the C-terminal poly(His)_10_-tag, a one-step IMAC purification was applied to get relative pure proteins from both P-CF and D-CF mode samples (Fig. 3). Binding of mAQP4 M23 to the IMAC column was relatively strong in the analysed detergents Fos-12 (1%), Fos-16 (2%), LPPG (1%) and Brij-35 (0.2%) and no elution was detected below 100 mM imidazol. Most impurities were washed off with 80 mM imidazol and the bound protein was eluted with 300 mM imidazol.

The SDS-PAGE indicates a prominent 30 kDa signal as the mAQP4 M23 monomer. An additional protein band at 66 kDa detected by immunoblotting could correspond to mAQP4 M23 dimeric complexes (Fig. 3). After immobilization of mAQP4 M23 to the Co^2+^ loaded IMAC column, detergents used for the initial solubilization of mAQP4 M23 could be exchanged by secondary detergents. Only a limited number of detergents are suitable for D-CF expression or for resolubilization after P-CF expression. While the D-CF mode requires very mild detergents, for the resolubilization of P-CF produced precipitates only relatively harsh detergents are useful. Those detergents used for the initial MP solubilization might not be optimal for subsequent assays and exchange against a second probably better suitable detergent could be beneficial. To test the possibility of primary detergent substitution of mAQP4 M23, Brij-35 in the proteomicelles of D-CF expressed mAQP4 M23 was exchanged to DDM (N-dodecyl-b-D-maltoside) (0.05%) or Fos-12 (0.05%), respectively. Fos-12 in the proteomicelles of P-CF expressed mAQP4 M23 was furthermore exchanged to DDM (0.05%). The detergent exchange from Brij-35 to Fos-12 resulted in apparent aggregation and precipitation of mAQP4 M23, while the protein remained soluble after the substitution with DDM from either Brij-35 or Fos-12. After elution of the resulting proteomicelles from the IMAC column, the concentrations of mAQP4 M23 in the peak fractions were determined in the range of 0.7–1 mg/ml.

Reconstitution of mAQP4 M23 into lipid bilayers is a prerequisite for the functional characterization of its water channel activity. The purified mAQP4 M23 was reconstituted into liposomes by following a previously published protocol [25]. The final concentration of mAQP4 M23 in reconstitution mixtures was approx. 100 µg/ml. Initial attempts to destabilize preformed liposomes (4 mg/ml) with DDM (0.04%) were not successful, which led to very low reconstitution rates or in even empty liposomes. When lipid concentration was increased to 6 mg/ml and DDM was replaced by Triton X-100 (0.36%), the mAQP4 M23 reconstitution rate was significantly increased. The prepared proteoliposomes could be stored at 4°C for up to one week. Extended storage as well as freezing in liquid nitrogen resulted in completely inactive proteoliposomes.

### Water channel activity of cell-free expressed mAQP4 M23

**Figure 4:**
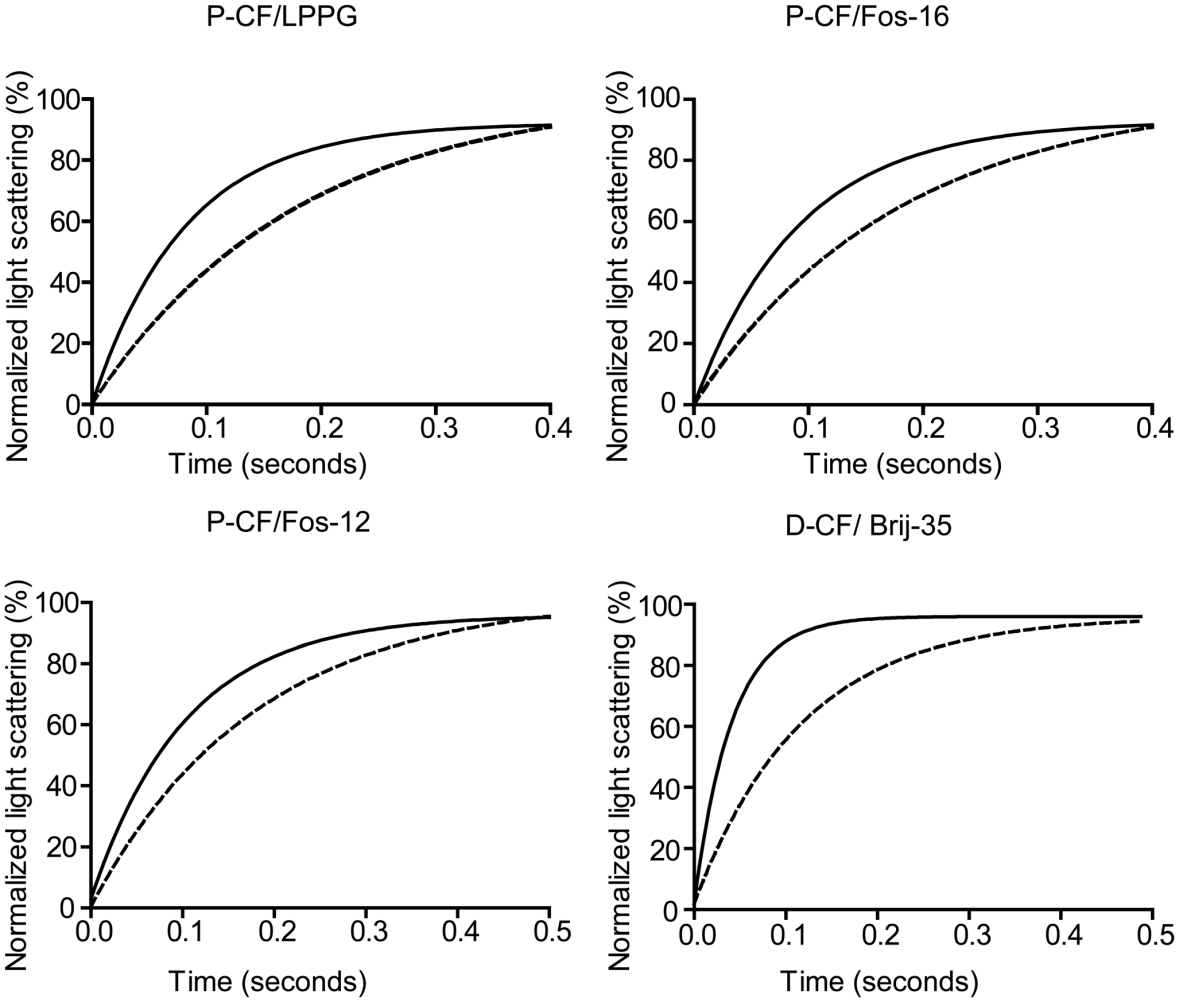
Water transport activity of P-CF and D-CF mode produced mAQP4 M23. Precipitates of P-CF produced mAQP4 M23 were resolubilized in the indicated detergents. The solubilized proteins were purified by Co^2+^-NTA chromatography, the initial detergent exchanged to 0.05% DDM and the samples were reconstituted into *E. coli* polar lipids. Water transport activity was determined by stopped-flow light scattering measurements of mAQP4 M23 proteoliposomes at 10°C. A 200 mM osmotic gradient was established by rapidly mixing vesicles suspended in reconstitution buffer with an equal volume of reconstitution buffer +400 mM sucrose. Data represent the average of three independent measurements. Fitted curves of mAQP4 M23 proteoliposome light scattering are shown. Solid line, mAQP4 M23 proteoliposomes; Dashed line, E. coli polar lipid empty liposomes.

Water channel activity of reconstituted mAQP4 M23 samples obtained from either P-CF or D-CF expression mode was analysed in a 100 µl reaction mixture, which was composed of 50 µl proteoliposomes and 50 µl reconstitution buffer with a final sucrose concentration of 200 mM. Reconstituted mAQP4 M23 proteoliposome and control empty liposomes were quickly mixed with the high osmotic reconstitution buffer by stopped-flow equipment at 10°C. The change of liposome volume as the result of water channel activity of inserted mAQP4 M23 was measured by light scattering at λ = 436 nm (Fig. 4). All mAQP4 M23 samples obtained from two different expression modes and primarily solubilized in four different detergents were analysed at 10°C. In all cases, the proteoliposomes had presented a higher water channel activity comparing with the control liposomes, which clearly suggested the functional reconstitution of mAQP4 M23 (Fig. 4). The *P*_f_ values of P-CF produced mAQP4 M23 samples resolubilized in Fos-12, Fos-16 and LPPG were determined as 54.4±2.5 µm/s, 49.4±2.0 µm/s and 50.7±2.7 µm/s, respectively. The *P*_f_ value obtained from D-CF produced mAQP4 M23 in Brij-35 was 133.1±5.6 µm/s. All control liposomes had an average *P*_f_ value of 25.5±1.6 µm/s.

**Figure 5:**
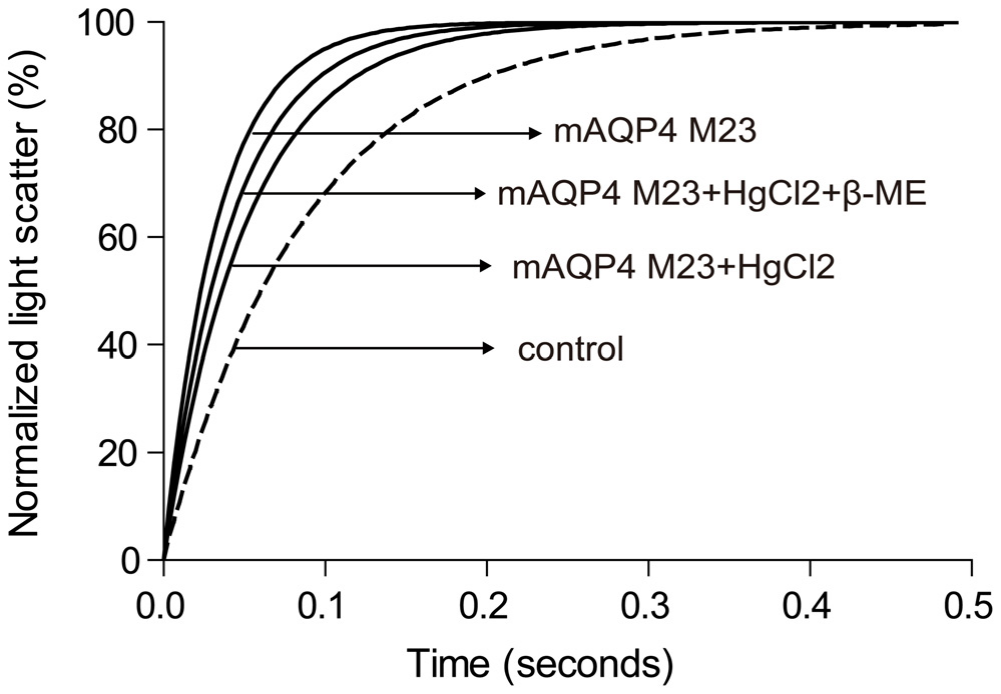
Specific inhibition of mAQP4 M23 water transport. Proteoliposomes containing D-CF produced mAQP4 M23 were treated with 300 µM HgCl_2_ for 5 min. at 23°C. For the recovery of the function of mAQP4 M23 function, 2 mM β-mercaptoethanol (β-ME) was added and incubated 10 min. at 23°C after incubation with HgCl_2_. Empty liposomes with and without treatment by HgCl2 were used as control and showed identical curves (dashed line).

**Figure 6:**
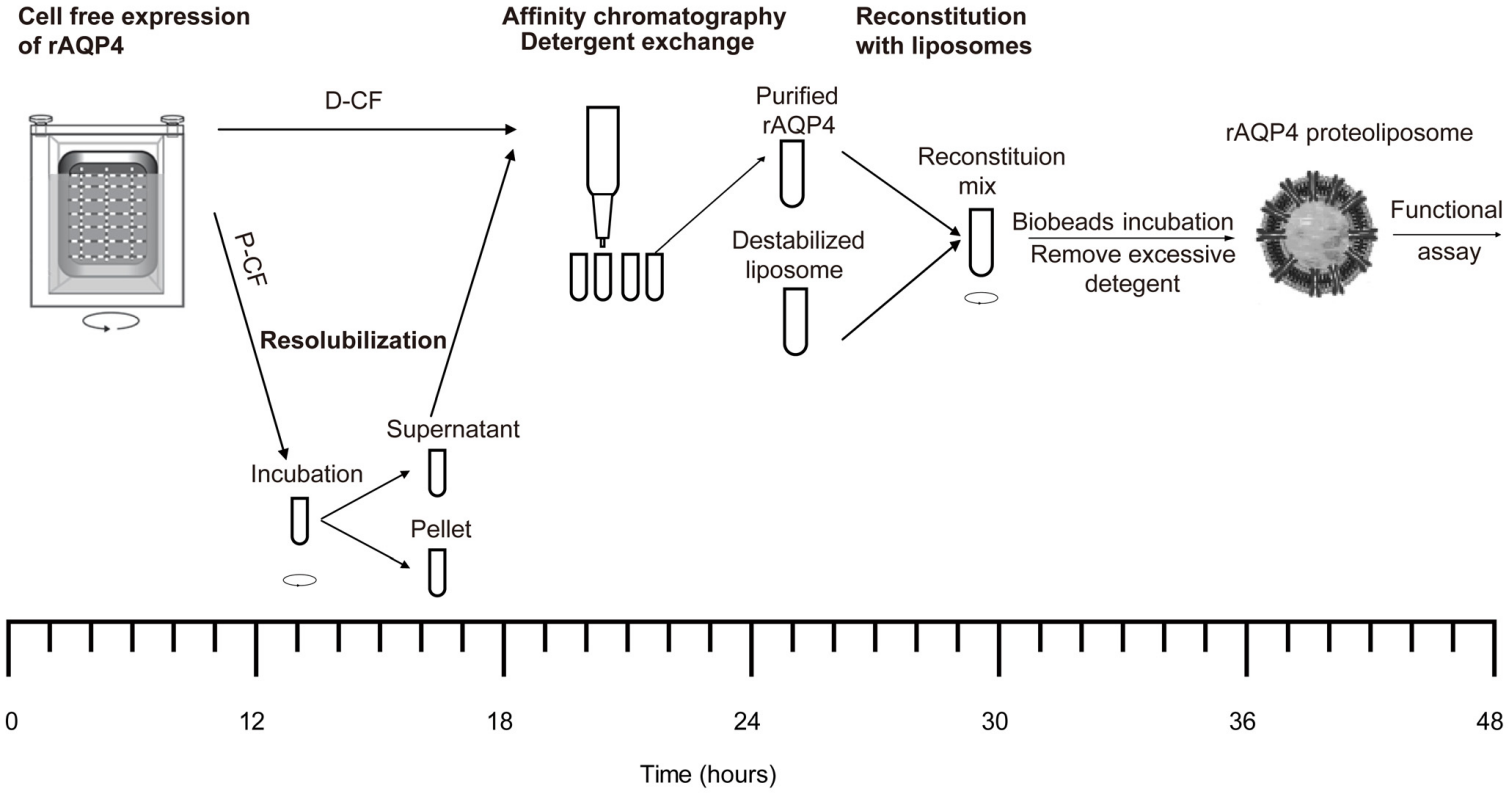
Flow-chart of mAQP4 M23 production by CF expression. The complete process from expression to functional analysis is finished within 2 days.

The water channel activity of aquaporins can be inhibited by the binding of HgCl_2_ to essential cysteine residues in the protein [26]. Pre-incubation of proteoliposomes containing D-CF produced mAQP4 M23 with 300 µM HgCl_2_ at 25°C for 5 min resulted in a clear reduction of the water channel activity from 158.3±3.0 µm/s to 103.8±2.7 µm/s (Fig. 5). The inhibition was partially reversible by treatment with ß-mercaptoethanol, which was presumably due to the regeneration of the essential thiol-residues, and the related *P*_f_ value was recovered to 126.0±2.8 µm/s. The *P*_f_ value of empty liposomes was 55.9±3.7 µm/s, with or without the treatment of HgCl_2_. The established complete process for the preparative scale CF production of mAQP4 M23 from expression to functionally active protein therefore takes no more than 2 days (Fig. 6).

## Discussion

Aquaporins are a ubiquitous class of MPs present in prokaryotes and eukaryotes that provide the cellular gatekeepers for water as well as for other small molecules such as glycerol. In human, 13 different and tissue specific AQPs are responsible for transport mechanisms and showed considerable clinical relevance [27]. AQP4 is expressed in the brain and thought to be primarily responsible for cerebral water homeostasis [28]. This protein is an important central regulator of cerebrospinal fluid which has to be very tightly controlled in order to prevent intracranial pressure resulting in compression of brain tissue, neurological disorders and even cell death. The two isoforms of AQP4 resulting from two optional translation initiation sites at methionine M1 or M23 are very unique. The shorter M23 isoform favors larger array formation in distinct tissues based on improved intermolecular contacts [29], [30]. Several structural information of AQPs has already been obtained by X-ray crystallization. The abundant AQP0 and AQP1 could be isolated in sufficient amounts from natural tissues whereas AQP4 and AQP5 were heterologously expressed in yeast cells [31], [32], [33], [34]. However, obtaining sufficient amounts of functional AQP samples still remains challenging and hampers their detailed molecular study. In addition, developing and screening for therapeutic drugs targeting on AQP4 would be valuable in addressing damages caused by stroke, edema, epilepsy and other CNS disorders.

This report demonstrated the first example for the preparative scale CF production of a functional eukaryotic AQP. Expression of rAQP4 that is approx. 92% identical to the mouse AQP4 has been approached in conventional *in vivo* systems before. But the expression in *E. coli* was not successful, and only a few µg of rAQP4 per liter of culture could be obtained from *Pichia pastoris* [35]. Best expression rates of up to 3 mg/l were obtained in Sf9 cell cultures after 72 hours infection [8], [35]. Summarizing from recent reports associated with AQP production, up to 15 mg/l hAQP4 could be produced in *P. pastoris* [34]; 3–5 mg/l of *Methanothermobacter marburgensis* AQPM was produced in *E. coli* [36]; 0.5 mg/l of human AQP2 was expressed in insect cells [37]; 9–13 mg/l of *E. coli* AQPZ was produced in *E. coli* [38] and 25 mg/L of spinach AQP-PM28A was produced in *P. pastoris* [39]. It was further suggested that construction of large fusion proteins may further improve the expression of AQPs [40]. However, most of AQPs are still difficult to produce, and solublization of overproduced AQPs from cellular membrane remains a challenging task. With the established CF expression protocol in this report, we were able to generate soluble and functional mAQP4 M23 within 24 hours. In addition to the considerably shortened expression time, the downstream purification process is significantly faster with the application of affinity purification column. The handling volumes by using CF expression technology are much smaller and expression rates of mg per ml can be achieved if compared with mg per litres with *in vivo* expression systems. This strategy will therefore dramatically accelerate the preparation of bioactive AQPs for both functional and structural assays.

The *E. coli* AQPZ was previously synthesized in a similar CF system using the batch configuration [41]. The protein was directly synthesized into preformed artificial liposomes that have been added into the reaction, resulting into proteoliposomes. In this study, we have used two different CF expression modes that both provided solubilized mAQP4 M23 samples, representing the preferred samples for structural analysis. In the P-CF mode, the protein was first precipitated and then post-translationally solubilized by detergents. In the D-CF mode, the mAQP4 M23 was co-translationally solubilized by detergents supplemented into the CF reaction. The results of water channel activity assays have indicated that both CF expression modes could produce functionally folded mAQP4 M23, while higher *P*_f_ values were obtained from D-CF samples. Osmotic water permeability values (*P*_f_) in reconstituted mAQP4 M23 proteoliposomes were calculated according to the results derived from light scattering assays and can be compared with corresponding values in the literature. The measured *P*_f_ values of ∼133 µm/s for mAQP4 M23 produced by D-CF at 10°C are comparable with reported *P*_f_ values of 28 µm/s for AQPZ at 6.5°C [1], *P*_f_ values of 57µm/s for AQPM at 4°C [36], and *P*_f_ values of 112 µm/s for rAQP4 expressed in yeast at 10°C [26]. Considering high variations in reconstitution rates and the assay temperature bias, our data obtained from the CF expressed mAQP4 M23 are in good agreement with the above mentioned *P*_f_ values. The lower *P*_f_ values of the P-CF produced samples at approx. 50 µm/s might also be a result of different reconstitution rates as different detergents were used for mAQP4 M23 solubilization.

Several higher *P*_f_ values of approx 200 µm/s of mAQP4 M23 are reported in the literature [42]. However, one should notice that most high *P*_f_ values were obtained by *in vivo* functional assays [42]. *P*_f_ values calculated by *in vitro* assays after reconstitution into artificial liposomes were usually below or around 100 µm/s, e.g. 89 µm/s [43]) or 117 µm/s [26]. In addition, lower *P*_f_ values (around 75 µm/s) were further observed in other *in vivo* measurements [44]. While there still potential exists for quality optimization of CF produced AQP in future, the functional parameter *P*_f_ clearly appears to be influenced by a number of factors which have to be considered if different samples were compared.

In the present work, two different CF expression approaches were developed that obtained sufficient amounts of functional eukaryotic AQPs within a short time. The presented protocols could be useful for structural characterization as well as for industrial applications of water filtration such as proposed by Swartz 2006 [9]. Lipid bilayers with functional AQP could be immobilized on top of porous supports, e.g. regenerated cellulose. With the specificity and high permeability of the selected AQP, only water can pass through the membrane while contaminants like ions and other molecules were not transported. This prototype could therefore provide an alternative way for water filtration. However, the requirement of large amounts of functional AQPs is the key bottleneck of this application.

## Materials and Methods

### Construction of the mAQP4 M23 expression plasmid

The 903 bp mAQP4 M23 gene was amplified from the plasmid T-vector-AQP4 M23 (obtained from Dongbei Normal University, China). The following two primers were used: 5′-GGAATTC**CATATG**- GTGGCTTTCAAAGG-3′ and 5′-GCCCG**CTCGAG** TACGGAAGACAAT-3′. The *Nde*I and *Xho*I restriction sites used for cloning are shown in bold. The restricted PCR products were ligated into a modified vector pIVEX 2.3d (Roche Diagnostics, Penzberg, Germany) containing a poly(His)_10_-tag. DNA templates used for CF expression were isolated from the resulting plasmid pIVEX2.3d-AQP4 M23 using commercial kits (Qiagen, Hilden, Germany).

### Cell free expression

Proteins were produced in CECF systems starting from previously described protocols [18], [19]. CF extracts were prepared from *E. coli* strain A19. Analytical scale reactions for the conditional optimization were performed in home-made Mini-reactors with membranes of regenerated cellulose and a molecular weight cut-off of 14 kDa [45]. The RM volume was 55 µl with a RM∶ FM ratio of 1∶15. The Mini-reactors were incubated in 24-well microplates holding the FM. Preparative scale reactions were carried out in home-made Maxi-reactors [45] in 1 ml of RM volume with a RM∶ FM ratio of 1∶17. As RM container, commercially available Slide-A-Lyzer were used (Pierce, Bonn, Germany). Either Mini-reactor or Maxi-reactor was incubated at 30°C for approx. 20 hrs with gentle shaking. For D-CF expression mode, the appropriate detergents were supplied into the reaction at certain final concentrations: Brij-35 (0.1%), Digitonin (0.4%), Triton X-100 (0.1%), Tyloxapol (0.05%). Triton X-100 was obtained from Merck Biosciences, Darmstadt, Germany; Brij-35, Digitonin and Tyloxapol were obtained from Sigma, Taufkirchen, Germany; LMPG and LPPG were obtained from Avanti Polar Lipids, Alabaster, Alabama, USA; Fos-12 and Fos-16 were obtained from Affymetrix® Anatrace, High Wycombe, United Kingdom; and DDM was obtained from AppliChem, Darmstadt, Germany.

### Liposome preparation

*E. coli* polar lipids were purchased from Avanti Polar Lipids, Inc. (Alabaster, AL). Lipid mixtures solubilized in chloroform were first transferred to a round bottom flask. A thin lipid film was formed by evaporating the chloroform under a nitrogen stream and placing the flask in a vacuum chamber for overnight. The lipids were then reconstituted in 1 ml of assay buffer (100 mM MOPS-KOH, pH 7.5) to a final concentration of 20 mg/ml by vortexing for 15 min to form multilamellar vesicles. The multilamellar vesicle solution was passed at least 21 times through an Avanti Polar Lipids mini extruder holding a 200 nm Whatman polycarbonate membrane filter (Florham Park, NJ) sandwiched with two filter supports on each side. The resulting unilamellar liposome solution was used for mAQP4 M23 reconstitution.

### Protein purification and reconstitution

The CF expressed mAQP4 M23 was purified in one step by imobilized metal-chelated affinity chromatography (IMAC). 1 ml of either D-CF soluble expressed mAQP4 M23 or P-CF resolubilized mAQP4 M23 was mixed with 300 µl of Co^2+^ loaded NTA resin slurry (Qiagen, Hilden, Germany). The mixture was diluted 10-fold with column buffer (20 mM Tris-HCl, pH 7.8, 300 mM NaCl, 20 mM imidazole, 0.05% DDM or 0.05% Fos-12) and incubated at 4°C for overnight with gently shaking. The mixture was poured into an empty column and washed at gravity flow rate of ten column volumes of column buffer supplemented with 80 mM imidazole. The bound mAQP4 M23 was then eluted with column buffer supplemented with 300 mM imidazole.

IMAC purified mAQP4 M23 protein was reconstituted into liposomes composed of *E. coli* polar lipids (Avanti Polar Lipids, Alabaster, AL, U.S.A.) by modification of a previously published protocol [25]. Briefly, a reconstitution mixture was prepared in a microtube at room temperature by sequentially adding reconstitution buffer (100 mM Mops, pH 7.5), 10% (v/w) Triton X-100 (final concentration 4 mM), 20 mg/ml preformed liposomes (final concentration 4 mg/ml), and 100 µg/ml purified mAQP4 M23. The reconstitution mixture was incubated at room temperature with gently shaking for 30 min. The detergent was removed by SM-2 beads (Bio-Rad, München, Germany) according to the manual. Finally, the liquid reconstitution mixture was sent to ultracentrifugation at 500,000 g for 45 min. Then, the pellet was washed again with reconstitution buffer. After wash step, the proteoliposome solution was ultracentrifugated again and finally resuspended in 1.6 ml reconstitution buffer.

### Water channel activity assay

Water permeability was measured by 90 degree light scattering at 436 nm in a stopped-flow spectrophotometer (SFM 300, BioLogic). Before measurement, the proteoliposomes were extruded once through a 200 nm membrane filter for homogenization. Proteoliposomes suspended in reconstitution buffer were quickly mixed with equal volumes of a hyper-osmotic solution (reconstitution buffer with 400 mM sucrose). Because sucrose does not penetrate into the proteoliposomes, the applied osmotic gradient initiates a water efflux. The resulting shrinking of the liposomes can be recorded by light scattering analysis. Data were fitted to an exponential rise equation, and the initial shrinkage rate (*k*) was determined by the average of at least three fitted equations. The water permeability factor *P*_f_ of the proteoliposome samples was calculated as described previously [1] using the equation:
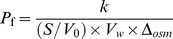
where S/V_0_ is the vesicle's initial surface to volume ratio, V*_w_* the partial molar volume of water (18 cm^3^ mol^−1^), and 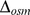 the osmotic driving force. The S/V_0_ was calculated by determining the diameter of the proteoliposomes using dynamic light scattering (ZetaPlus particle sizing software 2.27). The diameter of the proteoliposomes and empty liposomes were determined to be 113 nm. The 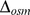 was 200 mM in this case.

Here one specific example was given as follows:

We take the D-CF reconstituted proteoliposome at 10°C as an example. *k* were obtained by three curve fitting to be 24.42, 25.33, 26.57. Then S/V_0_ was 5.31×10^7^ m^−1^. So the final *P*_f_ were 127.75 µm/s, 133.0 µm/s, 139 µm/s, respectively. Then the mean value and SD was given as 133.1±5.6 µm/s.
